# Fruit Self-Thinning: A Trait to Consider for Genetic Improvement of Apple Tree

**DOI:** 10.1371/journal.pone.0091016

**Published:** 2014-03-13

**Authors:** Jean-Marc Celton, Jean-Jacques Kelner, Sébastien Martinez, Abdel Bechti, Amina Khelifi Touhami, Marie José James, Charles-Eric Durel, François Laurens, Evelyne Costes

**Affiliations:** 1 Institut National de la Recherche Agronomique (INRA), UMR 1334, AGAP CIRAD-INRA-Montpellier SupAgro Team «Architecture et Fonctionnement des Espèces Fruitières», Montpellier, France; 2 Pépinières et Roseraies G. Delbard, Commentry, France; 3 Institut National de la Recherche Agronomique (INRA), UMR1345 Institut de Recherche en Horticulture et Semences (IRHS), AgroCampus-Ouest, Université d’Angers, SFR 4207 QUASAV, Beaucouzé, France; United States Department of Agriculture, United States of America

## Abstract

In apple (*Malus*×*domestica* Borkh), as in many fruiting crops, fruit maintenance vs abscission is a major criteria for production profitability. Growers routinely make use of chemical thinning agents to control total fruit load. However, serious threats for the environment lead to the demand for new apple cultivars with self-thinning properties. In this project, we studied the genetic determinism of this trait using a F_1_ progeny derived from the cross between the hybrid INRA X3263, assumed to possess the self-thinning trait, and the cultivar ‘Belrène’. Both counting and percentage variables were considered to capture the fruiting behaviour on different shoot types and over three consecutive years. Besides low to moderate but significant genetic effects, mixed models showed considerable effects of the year and the shoot type, as well as an interaction effect. Year effect resulted mainly from biennial fruiting. Eight Quantitative Trait Locus (QTL) were detected on several linkage groups (LG), either independent or specific of the year of observation or the shoot type. The QTL with highest LOD value was located on the top third of LG10. The screening of three QTL zones for candidate genes revealed a list of transcription factors and genes involved in fruit nutrition, xylem differentiation, plant responses to starvation and organ abscission that open new avenues for further molecular investigations. The detailed phenotyping performed revealed the dependency between the self-thinning trait and the fruiting status of the trees. Despite a moderate genetic control of the self-thinning trait, QTL and candidate genes were identified which will need further analyses involving other progenies and molecular investigations.

## Introduction

Organ abscission is a natural process that allows plants to remove damaged, senescent or mature organs. It results from the development of abscission zones in each organ, even though only one zone is activated at each specific developmental stage [1], [2], [3]. Fruit abscission has been particularly studied in a number of species such as tomato, grape, stone and pome fruits, because of its importance in determining fruit crop quantity and quality. In apple, fruit abscission occurs at three particular developmental stages, first a few days after anthesis, second in June before the beginning of exponential fruit growth, and third before ripening [4], [3]. Because flowers and fruits are formed in clusters located on terminal positions of the shoots, fruit drop involves, in addition to competition among inflorescences, and between inflorescences and vegetative shoot growth, a competition among developing fruits within a cluster [5], [6]. This competition has been described as a consequence of the relative position of the fruits within the cluster, with the terminal flower (also called “king flower”) being dominant [7] [3] [8]. The nutritional status of the young fruits, through the level of sucrose in the pedicel [9], as well as auxin and GA regulation and transport [3] [8] have been considered as factors involved in young fruit drop. In recent experiments, the molecular signatures related to fruit abscission induced by thinning chemicals have confirmed the involvement of a cross-talk between the nutritional status of the fruit and hormonal signalling in abscission zone activation [8] [10] [11] [12]. According to these authors, unfavourable nutritional conditions and sugar availability perceived by the young developing fruits induce simultaneously an up-regulation of ABA and ethylene with a down-regulation of GA signalling pathways. Thus, we hypothesize that within inflorescences lateral fruitlets may develop poorly due to unfavourable nutritional conditions, and because of the reduced sink they represent, their hormonal production and perception may be altered. This change in hormonal balance may in turn activate the development of an abscission zone, as well as a number of cell-wall degradation enzymes, such as cellulase, polygalacturonase or glycolases [11] [13] [14] [15], leading to fruitlet drop several days later.

In fruit tree industry, considering the huge amount of flowers or inflorescences that a fruit tree can bear, fruit load control has received particular attention. Indeed, an excess of fruits with respect to vegetative growth may lead to low fruit size and to irregular or biennal bearing in many perennial crops, particularly in apple, pear, plum, olive, and Citrus [16]. Thinning methods are thus widely used to promote fruit abscission and control fruit load [17], [18], [19] [1]. In apple, chemical thinning is commonly applied up to 30 days after full bloom, this period being considered optimal because fruit to fruit trophic competition and the detrimental effect of fruit on floral initiation are still low. However, the effect of thinning treatments is uncertain and largely depends on the cultivar and environmental conditions. Moreover, thinning agents such as the benzyladenine (BA) or the Naphtaleneacetic acid (NAA) may present a threat for the environment and their use is being restricted. This leads to the demand for alternative strategies among which the selection of new cultivars with self-thinning properties.

From a genetic point of view, most of the processes involved in yield determination vary greatly among cultivars. Varietal differences have been reported in the floribundity and propensity to regular bearing in the apple tree [20] [21] [22]. Spurs mutants with short internodes and lateral branching have an enhanced precocity and a greater fruit-setting ability [23], [24], even though they tend to be alternate bearing [25]. Additional evidence of the impact of vegetative shoot length on terminal bud floral induction and fruit setting has been provided, supporting the assumption of within tree variation of fruit set [5] [26]. Describing the genetic variability of architectural traits among cultivars and progenies, [20] noticed several cultivars and hybrids that exhibited a fruit self-thinning behaviour. These genotypes were mentioned as naturally maintaining one fruit per inflorescence after fruit set and were introduced as parents in several crosses at both Bordeaux and Angers INRA stations (France). Despite this strong hypothesis of genetic transmission of the self-thinning trait, no study was performed so far to confirm its genetic determinism. Natural variation of fruit abscission has been reported [27], but this study focused on the last developmental stages before ripening rather than on fruit abscission just after anthesis. In the present study, in addition to genetic control, we hypothesized a possible influence of crop load on the ability of the tree to self-thin the fruits because of the role of nutritional status of fruits in the natural drop. We thus investigated the genetic determinism of the self-thinning trait with respect to (i) its stability over years and annual variation of tree fruit load and (ii) within-tree trait variation resulting from different axis types bearing fruits. A detailed phenotyping of a progeny derived from an INRA hybrid and a QTL analysis were performed in order to identify the genomic regions involved in the genetic variation of this trait.

## Materials and Methods

### Plant Material

The F1 progeny under study is derived from a cross between the hybrid X3263 and the cultivar ‘Belrène’. The parents were initially chosen for their contrasted architectural traits, ‘Belrène’ exhibiting an erected tree habit (type II according to Lespinasse’s classification [28]) while X3263 is considered to have an intermediate growth habit (type III). This hybrid was bread at the INRA station of Angers and derived from a cross between ‘Red Winter’ and X3177, the latter being itself a hybrid derived from a cross between ‘Idared’ and ‘Prima’. X3263 hybrid was described as not sensitive to alternate bearing and exhibiting self-thinning trait (Y. Lespinasse, personal communication).

The segregating population is composed of 324 trees, of which 50 were randomly selected to produce replicates. Trees were grafted onto ‘Pajam 1’ apple rootstocks and planted in 2005 at a single location, the Melgueil INRA Montpellier experimental station. All hybrid trees as well as the parents and grand-parents present in the orchard were phenotyped, and 271 genotypes, including those with two replicates, were used for the linkage map construction. Because of alternate bearing behaviour, the number of trees observed varied depending on the year. Thus, 286 hybrid trees were observed in 2008, which corresponded to the first year of flowering of the progeny, 296 were observed in 2009 and 285 in 2010.

### Phenotyping

Fruit set was recorded on inflorescences born on two branches per tree. These branches, located along the trunks, had developed in the same year and were chosen as comparable as possible in terms of development. Along each branch, the successive years of growth were identified and the shoots born along those sections were classified in three types, depending on the length of growth units (GUs) that composed them as previously described in [29]: shoots were considered short when the length of all their GUs was less than 5 cm; they were classified as medium when at least one GU was more than 5 cm but less than 20 cm long; and shoots were considered long when at least one GU was more than 20 cm long.

Mid-April of the first observation year (2008), near full bloom, the number of inflorescences was counted along each selected branch, noting the bearing shoot category. The inflorescences that were axillary along the one-year-old shoot of the branch were also counted and classified as ‘axillary’. In June, about 70 days after full bloom (dafb) and after fruit set and natural thinning, the number of fruits per inflorescence that had set was counted. The following year (2009), the total number of inflorescences per shoot was recorded for the second time, at the same period as in the first year. The number of fruits per terminal inflorescence was counted twice: a first counting was performed in May (about 30 dafb) and the second in June (about 70 dafb). In the third year (2010), after having checked that the total number of inflorescences observed at 30 dafb and 70 dafb were highly correlated, the number of fruits set per inflorescence was recorded in May only (30 dafb).

Finally, we obtained a dataset that contained the following variables for three years: the total number of inflorescences per shoot type along a branch (NIn), the fruit set per branch that was estimated as the ratio between these two variables (NIn_s/NIn), and the number of fruits per terminal inflorescence (NFI). An additional variable was considered for each shoot type: the percentage of inflorescences with 1 fruit (%In_1fr) which corresponds to its ratio with respect to the total number of inflorescences (NIn_1fr/NIn).

### Data Analyses

All statistical analyses were performed using R software v3.0.2 [30], with lme4 package for mixed linear model estimation. Asreml software was used for the calculation of confidence interval on heritability values [31].

The normality of each variable distribution was checked. When not verified, the corresponding variable was transformed with a square root transformation before modelling (these variables are distinguished in Tables by the star symbol).

On all variables observed over three years, significance of the year, shoot type and genotype effects, and their first order interactions were estimated using the following model:
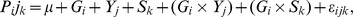
where P is the phenotypic value of the shoot *k* of the genotype *i* in year *j*, μ is the total average of the population, G_i_ is the effect of the genotype *i*, Y_j_ is the effect of the year *j*, S_k_ is the effect of the shoot type *k*, 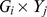 is the interaction between the genotype and year, 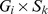 is the interaction between the genotype and shoot type, and ε_ijk_ is the residual error.

Mixed linear models were built for each variable, considering the year (Y) and shoot type (S) as fixed effects, the genotype (G) and the interactions with the genotype as random effects. The models were estimated with restricted maximum likelihood (REML) estimation method and effects to be included were selected on the basis of AIC and BIC (Akaike and Bayesian Information Criterion, respectively) minimization. For each trait, when G and interaction effects were included, the heritability of the mean genotype (h^2^) was estimated as the ratio between the genotypic variance and the total variance. Variables were considered as heritable if their h^2^ value was greater than 0.2 [32]. When an interaction 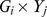 was included in the model the heritability was calculated as follows: 
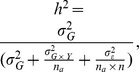
where n is the number of repetitions per genotype (2, in the present case), and n_a_ is the number of years (3 in the present case).

When the interaction 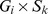 was also included, the calculation was:
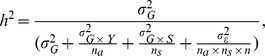
where n is the number of repetitions per genotype, n_a_ is the number of years and n_s_ is the number of shoot types (4 in the present case, corresponding to long, medium, short axillary shoots along the branches and inflorescences directly inserted in axillary positions along the 1-year-old section of the same branches).

Confidence interval for heritability values were estimated with asreml and deltamethod procedure.

### QTL Detection

Both parental genetic linkage maps and an integrated map of X3263×’Belrène’, previously developed [33], were used for QTL detection. These maps were constructed using 271 individuals with 83 SSR markers [34], [35] and 128 SNP markers [36]. A total of 211 genetic markers were mapped on the X3263×‘Belrène’ integrated genetic map, which covers 1068 cM over 17 LGs [33]. However, markers with similar alleles between parents (hk×hk) were discarded for QTL detection, and the map considered includes 186 markers.

QTL analysis was performed using MapQTL6 [37] on the mean genotypic values for all variables and on BLUP extracted from the linear mixed model for normally distributed variables only. QTLs were detected using the interval mapping (IM) (step size 1 cM) and multiple QTL mapping (MQM) functions. A QTL was declared significant if the maximum LOD score exceeded the genome wide LOD threshold calculated over 1000 permutations, with a mean error rate of 0.05. When a QTL was declared significant after IM, the nearest marker to the LOD peak was selected as co-factor for MQM. Each QTL was characterized by its LOD score, the percentage of phenotypic variation explained, and its confidence interval that corresponded to a LOD score drop of 1 or 2 on either side of the likelihood peak. The allelic effects were estimated for female and male additivity, and for dominance (Ollivier, 2002). Female (Af) and male (Am) additive effects were computed as 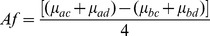 and 
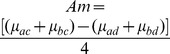
 respectively where 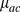, 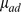, 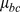, 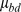 are estimated phenotypic means associated with each of the four possible genotypic classes deriving from a <ab×cd> cross. Additive effect represents the contrast between mean value per class including a vs b allele inherited from the female parent or c vs d allele inherited from the male parent, respectively. Dominance effect was computed as 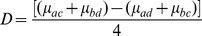 and corresponds to an interaction effect between female and male alleles. Length of SSR alleles (in bp) at loci associated with putative QTLs is indicated in Table S1.

QTLs were graphically displayed as bars next to the LG on which they were identified using MapChart version 2.0 [38]. When several QTLs were detected for a trait, a global model including all cofactors and their order 2 interactions, considered as fixed effects, was built to test for epistatic effects. The significant effects were selected on the basis of AIC and BIC criteria minimization. This modelling step allowed to estimate the global percentage of phenotypic variation (global R^2^) explained jointly by all the QTLs.

### Search for in silico Candidate Genes and miRNA Positioned under QTL Regions

Among the QTLs detected, we selected those which had a confidence interval less than 5 cM for a more in depth investigation of putative candidate genes. For this, markers flanking the QTL region were first retrieved from the reference apple genome, available at Genome Database for Rosaceaae (GDR) website (http://www.rosaceae.org/cgi-bin/gdr, 2013 Nov 1), and their position downloaded from Malus×domestica whole genome v1.0 Assembly & Annotation file “Malus_x_domestica.v1.0.markers.xls”. The sequences of markers not listed in this file were blasted against Apple genome v1.0 contigs with the GDR tool NCBI BLAST to recover their positions. The list of predicted apple genes located between the positions of the markers flanking the QTLs was extracted.

From the list obtained for each QTL region, we focussed particularly on transcription factors and genes which putative function suggest a role in development of floral organs, carbohydrates and water supply to the young fruits, and abscission processes. We also investigated the possible involvement of miRNA genes that could interact with TF or genes of interest in the selected QTL zones. For this, the genes sequences were submitted to the ‘Preloaded small RNAs/user-submitted transcripts’ tool of psRNATarget server [39] (http://plantgrn.noble.org/psRNATarget/?function=2) against the 207 sequences of miRBase (www.miRBase.org, Release 19, August 2012).

## Results

### Trait Variation Depending on the Observation Year and Shoot Type

**Figure 1:**
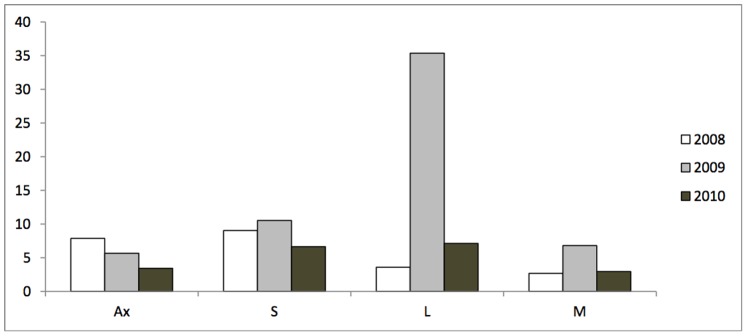
Mean number of inflorescences per shoot for each shoot type and year of observation in a F1 apple progeny derived from the cross ‘X3263’×‘Belrène’. Ax: Axillary inflorescences; S: Short shoots; M: Medium shoots; L: Long shoots.

Among the three years of observation, 2008 corresponded to the first year of fruit production. In the following year (2009), most trees exhibited a heavy crop load whereas the crop load was lower in 2010 (Figure 1). This behaviour corresponds to an irregular bearing, characterized by an “on” year in 2009 and “off” years in 2008 and 2010. The total number of inflorescences observed on all the sampled branches varied considerably between years, with about 10.000 inflorescences in 2008, 34.000 in 2009 and 6.000 inflorescences observed only in 2010. The alternate bearing was also apparent at the local scale, i.e. the mean number of inflorescences per shoot type was higher in the “on” year than in the “off” years on three shoot types (Figure 1). The difference between years was more pronounced on long shoots than on medium and short shoots, this latter being proportionally less represented in the “on” year (data not shown). However, the difference in mean number of inflorescences per shoot type between “on” and “off” years was not observed for inflorescences born in axillary position. In that case, the mean number of inflorescences slightly decreased over the three years, independently from the “on” or “off” status of the year.

**Figure 2:**
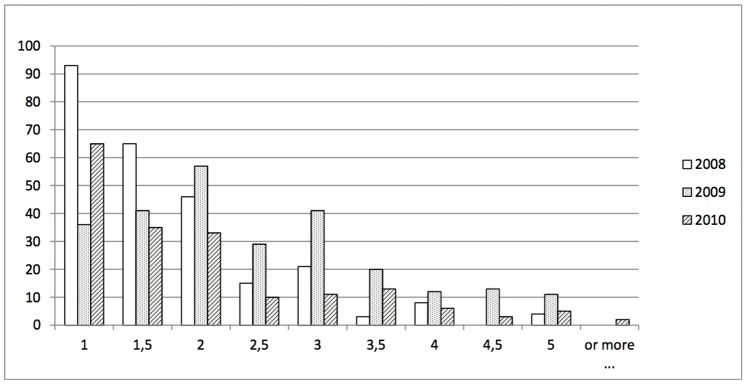
Distribution of the mean number of fruits per inflorescence with fruit set depending on the year of observation in a F1 hybrid apple tree population derived from the cross X3263×‘Belrène’. (See also the mean values observed each year for the two parents and grand-parents in Table 1).

Considering the inflorescences with fruit set only, the distribution of the mean number of fruits per inflorescence was L-shaped for the two “off” years, with a majority of inflorescences supporting one fruit and few inflorescences with a high number of fruits (Figure 2). However, we found more inflorescences with two fruits than a single fruit in the “on” year. As expected, ‘Belrène’ had a higher mean number of fruits per inflorescence than X3263, whatever the year (Table 1, Figure 2). It is remarkable that the parent X3263 had slightly more fruits per inflorescence than its own parent X3177 which number of fruits per inflorescence was close to 1 whatever the year and its bearing status (“on” or “off”). For all parents, grand-parents and progenies, the mean number of fruits per inflorescence was higher in the “on” year (2009) than in the two “off” years (2008 and 2010).

**Table 1:** Mean number of fruits per inflorescence with fruit set for each parent (Belrène and X3263) and two grand-parents (Red Winter and X3177), depending on the year.

	2008	2009	2010	All years
Belrène	2.92	3.49	2.17	2.87
X3263	1.82	2.01	1.53	1.77
Red Winter	1.80	2.47	2.76	2.31
X3177	1.03	1.36	1.32	1.25

Spearman correlation coefficient values indicated that the studied variables were correlated to each other (Table 2). The total number of inflorescences (NIn) was poorly correlated with the other variables. Interestingly, the slightly negative phenotypic correlation with fruitset (−0.47) was almost null for genotypic correlation, suggesting that antagonism between number of inflorescences and fruitset was entirely environmentally driven. Correlation between fruitset and the number of fruits per inflorescence was positive (0.37 and 0.53 for phenotypic and genotypic correlations respectively) whereas negative values were observed between fruitset and the percentage of inflorescences with one fruit (−0.35 and −0.52 for phenotypic and genotypic correlations respectively). The increase in absolute value when considering genotypic correlations is noticeable. Finally, the number of fruits per inflorescence was highly and negatively correlated to the percentage of inflorescences with 1 fruit (−0.86 and 0.87 for phenotypic and genotypic correlations respectively).

**Table 2:** Spearman phenotypic (below diagonal) and genotypic (above diagonal) correlation coefficients between the total number of inflorescences per branch (NIn), the fruit set, the number of fruit per inflorescence in terminal position (NFI), the percentage of inflorescences with 1 fruit (%In_1fr).

	NIn*	fruit_set	NFI*	%In_1fr
NIn*	1	0.02	0.08	−0.12
fruit_set	**−0.47**	1	**0.53**	**−0.52**
NFI*	0.02	0.37	1	**−0.87**
%In_1fr	0.11	−0.35	**−0.86**	1

Variables transformed with a square root transformation are indicated by * symbol. Significant coefficients are indicated in bold.

### Genetic and Fixed Effects

The mixed linear model with three factors revealed a significant random genetic effect for all the studied variables, except the total number of inflorescences per branch (Table 3). For all variables, both the year and shoot type and their interaction were included as fixed factors in the selected models. The relative influence of fixed effects can be analysed through the effect estimates on the mean values, which are expressed with the medium shoots in 2010 as reference (Table 4). This analysis confirms that NIn and NFI variables had lower mean values in 2008 and 2010 than in 2009, whereas the year effect was inverse on the percentage of inflorescences with 1 fruit (%In_1fr). Long shoots had higher mean values of total number of inflorescences (NIn), number of fruit per inflorescence (NFI) and fruit set than medium shoots, whereas inflorescences in axillary position had lower mean values for these variables. Interactions between years and shoot types also revealed that the total number of inflorescences (NIn) was lower in the “on” year (2009), compared to “off” years (2008 and 2010) for inflorescences in axillary position and located on short shoots whereas the inverse was observed for long shoots. The impact of these interactions on the mean values of the other variables was lower, even though significant.

**Table 3:** Effects of genotype (G), year (Y) and shoot type (ST) and their first-order interactions in the models selected for the total number of inflorescences per branch (NIn), the fruit set, the number of fruits per inflorescences (NFI), the percentage of inflorescences with one fruit (%In_1fr), in a F1 apple progeny derived from X3263×‘Belrène’ cross.

	Model selected		Variance estimates of random factors			h^2^	CI
Variable	Fixed effects	Random effects	G	G:Y	G:ST		
NIn*	Y+ST+YxST	G+GxY+GxST	–	23.10	23.422	–	–
Fruit set	Y+ST+YxST	G+GxY+GxST	3087	3304	395	0.66	0.61–0.70
NFI*	Y+ST+YxST	G+GxY+GxST	609.1	997.7	134.5	0.57	0.52–0.62
%In_1fr	Y+ST+YxST	G+GxY+GxST	165.7	297	59.7	0.52	0.48–0.56

Broad sense heritability values (h^2^) and their confidence interval (CI) are indicated for each variable. Variables transformed with a square root transformation are indicated by * symbol.

**Table 4:** Estimates of year (Y), shoot type (ST) and their first-order interactions in the models selected for the total number of inflorescences per branch (NIn), the fruit set, the number of fruits per inflorescences (NFI), the percentage of inflorescences with one fruit (%In_1fr), in a F1 apple progeny derived from X3263×‘Belrène’ cross.

Year _ shoot type	NIn*	Fruit set	NFI*	%In_1fr
2010 _ Medium	8.11	215.92	75.90	51.98
2008	−2.55	−31.04	−20.16	14.24
2009	5.65	23.66	25.18	−8.40
Axillary	−3.34	−38.15	−23.14	18.86
Short	0.43	−10.89	−5.35	6.78
Long	7.11	22.38	17.38	−17.72
2008 _ Axillary	5.33	−24.20	3.39	−1.46
2009 _ Axillary	−6.72	26.95	−4.18	−3.59
2008 _ Short	2.93	−2.38	1.54	−3.64
2009 _ Short	−4.01	4.01	−7.85	−0.06
2008 _ Long	−9.41	19.43	3.29	3.36
2009 _ Long	14.05	−25.85	9.49	4.33

For estimates, medium shoots in year 2010 is the reference. Variables transformed with a square root transformation are indicated by * symbol.

The selected models also included interactions between the genotype and the year and the shoot type factors for all the studied variables. Medium heritability values were obtained for the number of fruits per inflorescence and the percentage of inflorescences with one fruit with confidence interval between 0.48–0.56 and 0.52–0.62, respectively (Table 3). Fruit set exhibited a higher heritability with a confidence interval from 0.61 to 0.70.

### QTL Detection

**Figure 3:**
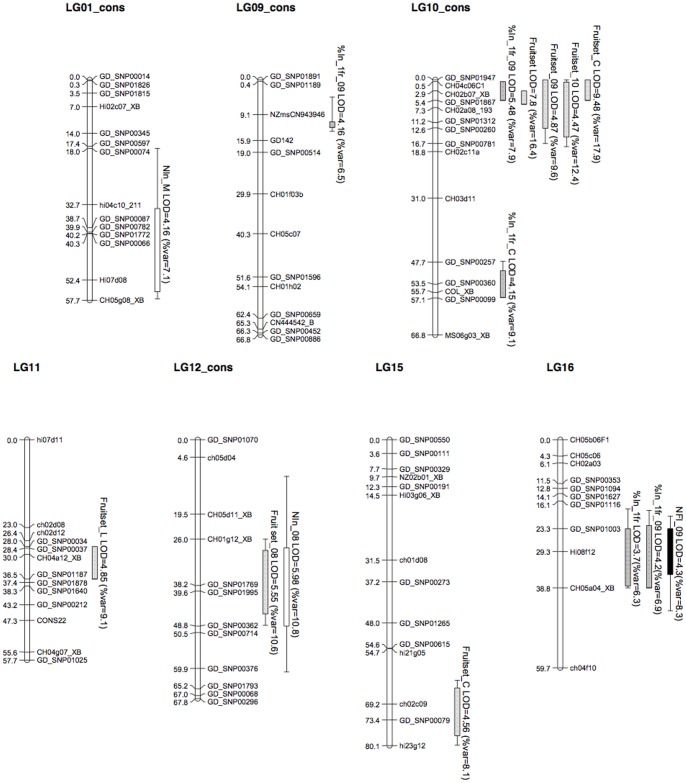
Genomic positions of the QTLs detected on the ‘X3263’×‘Belrène’ consensus map. QTLs are represented by boxes, in which bold lines represent the LOD–1 confidence interval and extended lines represent the LOD–2 confidence interval. Boxes representing QTLs for the number of inflorescences are white, those for the number of fruits per inflorescence are black, for the fruitset traits are pale grey, and for the percentage of inflorescences with 1 fruit are dark grey. For trait abbreviations, see Table 5.

Two QTLs were identified on the consensus genetic map for the mean genotypic values of the total number of inflorescences per shoot type or year (Table 5, Figure 3). A QTL located on LG12 was detected for the total number of inflorescences in 2008 (NIn_08), consistently with the significant GxY interaction (Table 3). This QTL explained 11% of the variance, and was characterized by a male additive effect, and was also detected on the male ‘Belrène’ parental map (with a LOD score of 3.4). A second QTL was detected for the total number of inflorescences per medium shoot (NIn_M) on LG1, consistently with the significant GxST interaction (Table 3). It explained 7% of the trait variance and was characterized by a female additive effect. This QTL was also identified in the female ‘X3263’ parental map (with a LOD score of 4.1).

**Table 5:** Parameters associated with the QTLs detected by interval mapping (IM) and multiple QTL mapping (MQM) for the genotypic mean values of the total number of inflorescences (NIn), the percentage of inflorescences with one fruit (%In_1fr), the number of fruits per terminal inflorescence (NFI) and fruit set, depending on the year and the shoot type (_08, _09, _C and _L for year 2008, 2008; short and long shoot respectively).

Trait	Method	LG^a^	Co_factor^b^	LOD^c^		Allelic effect d	*Af*	*Am*	*D*	%^e^
nbTinflo_08*	IM	12	GD_SNP01769	5.98	(4.0)	Am	−0.55	1.06	−0.15	10.8
nbTinflo_M*	IM	1	hi07d08	4.16	(4.0)	Af	−0.91	0.11	−0.14	7.1
%inflo_1fr	IM	16	Hi08f12	3.97	(3.9)	Am, D	0.02	−0.06	0.05	6.3
%inflo_1fr_09	MQM	10+	CH02b07_XB	5.48	(4.0)	Am, D	0.03	−0.09	−0.08	9.1
		16	Hi08f12	4.20		Am	0.04	−0.09	−0.01	6.9
		9	GD142	4.16		Af, D	−0.16	−0.07	0.24	6.5
%inflo_1fr_C	IM	10	COL_XB	4.15	(4.0)	Am	−0.06	−0.07	−0.07	7.9
nbfr_inflo_09*	IM	16	Hi08f12	4.33	(4.0)	Am	−0.13	0.30	−0.03	8.3
Fruitset	IM	10+	GD_SNP01867	7.54	(4.0)	Am, D	−0.05	0.07	0.07	12.4
Fruitset_08	IM	12+	GD_SNP01769	5.55	(4.1)	Af, Am	0.11	−0.09	0.02	10.6
Fruitset_09	IM	10+	GD_SNP01867	4.87	(4.1)	D	−0.04	0.06	0.09	9.6
Fruitset_10	IM	10	CH02b07_XB	4.47	(4.1)	Am, D	−0.06	0.09	0.09	12.4
Fruitset_C	MQM	10	CH02b07_XB	9.48	(4.0)	Am	−0.10	0.15	0.08	17.9
		15	ch02c09	4.56		D	0.05	−0.03	0.12	8.1
Fruitset_L	IM	11	CH04a12_XB	4.85	(3.9)	Af, Am	−0.07	0.05	−0.03	9,1

a Linkage Group.

b markers in QTL interval. used as cofactors in the MQM analysis.

c Maximum LOD score value with the considered threshold in brackets.

d Female (Af) and male (Am) additive effects computed as [(μac+μad)−(μbc+μbd)]/4 and [(μac+μbc)−(μad+μbd)]/4 respectively; dominance (D) and dominance effect computed as [(μac+μbd)−(μad+μbc)]/4. where μab. μad. μbc and μbd are estimated phenotypic means associated with each of the four possible genotypic classes ab. ac. ad and bd. deriving from a <ab×cd> cross.

e Percentage of phenotypic variation explained by the QTL.

+ QTL detected when using BLUP instead of mean genotypic value.

Variables initially transformed with a square root transformation are indicated by * symbol. Parameters of QTL detected when the Best Linear Unbiased Predictor (BLUP) of the genotypic effect were used, are indicated in italics.

Several QTL were detected for the percentage of the total number of inflorescences with one fruit. A close-to-significant QTL was detected on LG16 for this variable considered whatever the year and the shoot type. It explained a relatively low percentage of the variance (6.5%) and was characterized by both male and dominance effect. The same genomic region was found to control the percentage of inflorescences with one fruit in 2009, with similar characteristics. Two other QTLs were detected for this trait on LG9 and LG10, and explained 6.9 and 9.1% of the variance, respectively. The global model built with the three selected co-factors included a significant interaction between CH02b07_XB on LG10 and GD142 on LG9, and explained 30% of the total variance of the percentage of inflorescences with one fruit in 2009. Another QTL located on LG10 near the COL marker was detected for the percentage of inflorescences with one fruit on spurs. This QTL explained 7.9% of the variance and involved mainly a male additive effect even though it was not detected on the parental map.

No or few QTLs were detected for the number of fruits per inflorescence whatever the shoot type or the year considered. Only one QTL was identified for the number of fruits per terminal inflorescence in 2009 (NFI_09) on LG16, at a similar region than previously detected for inflorescences. This QTL explained 8% of the trait variability and was characterized by a male additive effect. It was also detected on the male parental map with a LOD score value of 3.4. By contrast, several QTL were detected when the fruit numbers were considered as a percentage, i.e. for fruit set related variables (Table 5, Fig. 3). A QTL was detected on the distal part of LG10 for fruit set whatever the year or the shoot type. It explained 12.4% of the variance and was characterized by a male additive and a dominance effect. This QTL was in the same genomic region as those detected for the percentage of inflorescence with one fruit in 2009 and was detected on ‘Belrène’ parental map with a LOD value of 3.0. Other QTLs were detected in the same region for fruit set in 2009 and 2010, and for fruit set of spurs. These QTL explained 9.6%, 12.4%, and 17.9% of the variance, respectively. The QTL for fruit set in 2009 was characterized by a dominant effect whereas that for fruit set in 2010 was characterized by both a male additive and dominance effects. The QTL for fruit set of spurs was characterized by a male additive effect and was also present on the parental map (LOD 4.0). Another QTL was detected for this trait on LG15, and explained 8.1% of the variance. Both QTL explained 13% of the variance of fruit set on spurs without interaction between the two corresponding co-factors (CH02b07_XB and ch02c09). For fruit set in 2008 a QTL was identified on LG12.It explained 12.6% of the variance and was characterized by both male and female additive effects. For fruit set on long shoots a QTL was located on LG11, and explained 9.1% of the variance. These two QTLs were also identified in the female parental map with a LOD score of 3.5 and 2.6 respectively (the genome wide threshold being 2.5).

### Screening for Putative Candidate Genes

Three QTL regions were selected and screened for putative candidate genes (gene list in Table 6 and Table S2). Two regions were located on LG10 and one on LG9. During this investigation, particular attention was paid to transcription factors and predicted genes involved in vascular tissues development and sugar transport, both of which processes may contribute to the maintenance or abscission of fruitlets (Table 6 and Supplementary Tables).

**Table 6:** List of the most remarkable annotated sequences in three QTL zones of interest, one on LG9 and two on LG10.

Category	Query ID	ScaffoldID	TranscriptStart	TranscriptLength	Match ID	Organism	Description	PercentIdentity	E-value	Nb ofcopiesin QTLzone	Total nbof copies inreferencegenome
TF	MDP0000366019	chr9	4405214	4745	AT3G02310.1	At	SEP2 (SEPALLATA 2); DNA binding/protein binding/transcription factor	65,02	2E-66	2	4
	MDP0000605482	chr9	4411551	3918	AT5G15800.1	At	SEP1 (SEPALLATA1); DNA binding/ transcription factor	82,19	7E-66	2	9
	MDP0000126997	chr9	5235116	451	AT2G03710.3	At	SEP4 (SEPALLATA 4); DNA binding/ transcription factor	90,16	3E-28	1	7
	MDP0000132738	chr9	4444708	561	AT5G60910.1	At	AGL8 (agamous-like 8); transcription factor	86,15	5E-27	1	5
	MDP0000143531	chr9	4509598	942	AT4G24540.1	At	AGL24 (AGAMOUS- LIKE 24); protein binding/proteinheterodimerization/ protein homodimerization/ sequence-specific DNA binding/transcription factor	67,21	2E-17	2	12
	MDP0000132959	chr9	5348078	546	AT2G37630.1	At	AS1 (ASYMMETRIC LEAVES 1); DNA binding/protein homodimerization/ transcription factor	64,1	7,00E-08	1	3
	MDP0000154734	chr9	677443	2714	AT5G13080.1	At	WRKY75; transcription factor	72,28	6E-39	2	7
Senescence	MDP0000633623	chr9	2246213	5883	AT5G14930.2	At	SAG101 (SENESCENCE- ASSOCIATED GENE 101); carboxylesterase/ triacylglycerol lipase	33,77	5E-73	7	18
	MDP0000187883	chr9	4656219	1005	AT5G15720.1	At	GLIP7; carboxylesterase/ lipase	38,08	5E-42	3	4
Glycosyl-transferases	MDP0000822876	chr9	1451274	1459	AT3G27540.1	At	glycosyl transferase family 17 protein	74,25	3E-167	2	3
Cellulosesynthases& Galacturonases	MDP0000314103	chr9	1496070	6493	AT5G05170.1	At	CEV1 (CONSTITUTIVE EXPRESSION OF VSP 1); cellulose synthase/transferase, transferring glycosyl groups	84,92	0	2	8
	MDP0000183140	chr9	2214322	2799	AT3G28180.1	At	ATCSLC04 (CELLULOSE- SYNTHASE LIKE C4); cellulose synthase/ transferase, transferring glycosyl groups	75,36	0	2	4
	MDP0000932170	chr9	2653142	1037	AT5G15050.1	At	glycosyltransferase family 14 protein/ core-2/I-branching enzyme family protein	71,7	5E-95	2	7
	MDP0000517518	chr9	2794153	2049	AT5G15870.1	At	glycosyl hydrolase family 81 protein	61,64	0	1	7
	MDP0000283839	chr9	3215719	4003	AT3G01180.1	At	AtSS2 (starch synthase 2); transferase, transferring glycosyl groups	65,59	0	1	2
	MDP0000152152	chr9	3802610	3365	AT5G15470.1	At	GAUT14 (Galacturonosyltransferase 14); polygalacturonate 4-alpha- galacturonosyltransferase/ transferase, transferring glycosyl groups/ transferase, transferring hexosyl groups	85,85	0	2	3
	MDP0000298545	chr9	4082535	612	AT3G02120.1	At	hydroxyproline-rich glycoprotein family protein	64,44	2E-11	1	3
	MDP0000394728	chr9	4386339	2317	AT5G15650.1	At	RGP2 (REVERSIBLY GLYCOSYLATED POLYPEPTIDE 2); transferase, transferring hexosyl groups	88,99	0	1	2
	MDP0000364776	chr9	4765572	502	AT3G02350.1	At	GAUT9 (Galacturonosyltransferase 9); polygalacturonate 4-alpha- galacturonosyltransferase/ transferase, transferring glycosyl groups/ transferase, transferring hexosyl groups	61,64	2E-13	2	3
	MDP0000148377	chr9	5010079	2076	AT5G15870.1	At	glycosyl hydrolase family 81 protein	71,56	0	1	7
Auxintransport	MDP0000286938	chr9	2484954	1708	AT3G28390.1	At	PGP18 (P- GLYCOPROTEIN 18); ATPase, coupled to transmembrane movement of substances	68,21	8E-39	1	9
	MDP0000592047	chr9	2487928	1309	AT3G28380.1	At	PGP17 (P- GLYCOPROTEIN 17); ATP binding/ATPase /ATPase, coupled to transmembrane movement of substances/nucleoside- triphosphatase/ nucleotide binding	64,1	2E-65	2	2
	MDP0000320690	chr9	3021773	6858	AT3G28860.1	At	ABCB19; ATPase, coupled to transmembrane movement of substances /auxin efflux transmembrane transporter	83,59	0	2	7
	MDP0000283840	chr9	3220552	2738	AT3G28970.1	At	AAR3 (antiauxin- resistant 3)	53,01	1E-80	1	2
Expansines	MDP0000432497	chr9	3249924	1097	AT2G03090.1	At	ATEXPA15 (ARABIDOPSIS THALIANA EXPANSIN A15)	77,73	4E-87	2	3
	MDP0000679320	chr9	3952913	354	AT5G39300.1	At	ATEXPA25 (ARABIDOPSIS THALIANA EXPANSIN A25)	45,24	1E-24	2	5
TF	MDP0000157175	chr10	5206681	201	AT1G22380.1	At	AtUGT85A3 (UDP-glucosyl transferase 85A3); glucuronosyltransferase /transcription factor/transferase, transferring glycosyl groups	41,79	2,00E-08	1	20
	MDP0000169115	chr10	5639948	1416	AT2G04890.1	At	SCL21 (SCARECROW-LIKE 21); transcription factor	32,99	1E-41	2	3
	MDP0000849944	chr10	5899220	1531	AT1G71930.1	At	VND7 (VASCULAR RELATED NAC-DOMAIN PROTEIN 7); transcription activator/transcription factor/transcription regulator	55,76	7E-88	1	6
	MDP0000913269	chr10	5837731	687	AT3G60390.1	At	HAT3 (HOMEOBOX-LEUCINE ZIPPER PROTEIN 3); transcription factor	80,77	6E-17	1	5
Senescence	MDP0000086659	chr10	5652509	1129	AT5G45890.1	At	SAG12 (SENESCENCE-ASSOCIATED GENE 12); cysteine-type peptidase	57,73	4E-112	1	28
Glycosyl_hydrolases& sucrosetransporters	MDP0000234500	chr10	6131258	762	AT1G30080.1	At	glycosyl hydrolase family 17 protein	45,57	8E-14	1	4
	MDP0000129284	chr10	6395682	1164	AT1G78800.1	At	glycosyl transferase family 1 protein	52,03	2E-29	3	11
	MDP0000137383	chr10	5601556	1507	AT1G71880.1	At	SUC1 (Sucrose-proton symporter 1); carbohydrate transmembrane transporter/sucrose:hydrogen symporter/sugar: hydrogen symporter	64,68	5E-141	1	3
	MDP0000774366	chr10	5610911	559	AT1G22710.1	At	SUC2 (SUCROSE-PROTON SYMPORTER 2); carbohydrate transmembrane transporter/sucrose transmembrane transporter/sucrose:hydrogen symporter/sugar: hydrogen symporter	77,01	2E-27	2	6
TF	MDP0000162468	chr10	26355418	1416	AT2G04890.1	At	SCL21 (SCARECROW-LIKE 21); transcription factor	32,99	1E-41	1	3
	MDP0000324166	chr10	26500109	7258	AT4G18960.1	At	AG (AGAMOUS); DNA binding/transcription factor	70,52	8E-99	1	3
	MDP0000582861	chr10	26774460	2108	AT1G71692.1	At	AGL12 (AGAMOUS-LIKE 12); transcription factor	38,1	8,00E-08	2	4
	MDP0000133918	chr10	27085544	1225	AT1G29280.1	At	WRKY65; transcription factor	52,23	7E-59	1	3
	MDP0000285490	chr10	27135441	2177	AT1G62360.1	At	STM (SHOOT MERISTEMLESS); transcription factor	79,09	2E-38	1	5
Trehalose &cellulosesynthases	MDP0000227381	chr10	26448598	5975	AT1G78580.1	At	ATTPS1 (TREHALOSE-6-PHOSPHATE SYNTHASE); alpha,alpha-trehalose-phosphate synthase (UDP-forming)/transferase, transferring glycosyl groups	72,53	0	1	10
	MDP0000265728	chr10	27049737	3142	AT1G06410.1	At	ATTPS7; alpha,alpha-trehalose-phosphate synthase (UDP-forming)/transferase, transferring glycosyl groups/trehalose-phosphatase	76,46	0	1	8
	MDP0000214413	chr10	27203159	5378	AT4G18780.1	At	IRX1 (IRREGULAR XYLEM 1); cellulose synthase/transferase, transferring glycosyl groups	81,95	0	2	9

The first genomic region investigated on LG10 was comprised between the markers CH02b07 and GD_SNP01867. It spanned the length of about 2 Mb and included 243 predicted genes. Within this zone, 17 transcription factors (TF) were identified, among which three were previously found to be involved in vascular tissues development and sugar transport in *Arabidopsis thaliana*. In particular, we identified a Vascular Related NAC-domain TF homolog to *Arabidopsis thaliana* VND7 (see Ath and MDP numbers in Table 6). Among the genes located within the QTL confidence interval we also identified a UDP-glucosyl transferase involved in the biosynthesis of poly-saccharides and two carbohydrate trans-membrane transporters (SUC1 and SUC2), the second one with two copies. Even though several SUC copies were present on almost all LGs, SUC1 had only three copies and SUC2 exhibited 6 copies along the apple reference genome.

In the same zone, we also found two copies of SCARCROW-LIKE 21 protein (SCL21). SCL genes belong to the large GRAS family and are involved in a number of developmental processes such as GA responses controlling flowering, shoot and root apex development or xylem patterning. A third copy of SCL21 was present in the second QTL zone on LG10, close to CO marker. These three copies are the only ones present along the apple genome for SCL21 gene.

The second QTL zone investigated on LG10 was comprised between the markers GD_SNP00360 and COL, and spanned 1.2 Mb. It contained 247 predicted gene sequences, including ten TF. Among these, we identified another predicted SCL21 TF located close to the COL marker. Together with the two SCL21 previously identified, these three copies are the only ones present along the apple genome. In addition this QTL region contained one copy of AGAMOUS and two copies of AGAMOUS-LIKE 12 (AGL12). Among the four copies of AGL12 found in the apple reference genome, two were located on LG8 and the two other ones were in LG10, included in the present QTL zone. We also identified a TF homologue to SHOOT MERISTEMLESS (STM): five copies were found on the apple reference genome, two on LG10, one copy contained in the QTL zone whereas the other one was slightly above.

Within the QTL confidence interval, we also identified WRKY65, a member of the large WRKY family. This family is involved in the complex regulation of senescence, especially in response to biotic and abiotic stress. Only three copies of WRKY65 were found in the reference genome on LG5, LG10 and LG15.

Several genes involved in sugar synthesis were also identified within this QTL. These include genes homologue to ATTPS1 and ATTPS7, two trehalose synthases, and IRX1 (also known as CESA8) involved in cellulose synthase. Seven other copies of IRX1 were present on the apple reference genome, 6 on LG5 and one on LG16.

Three Md-miRNAs were listed in the Predicted miRNA/Target Pairs analysis among the genes included in this LG10 QTL confidence interval: Md-miR390 (6 isogenes), Md-miR7124 (2 isogenes) and Md-miR482a-p (list in Table S3 and S4). The first miRNA has a cleavage function on MDP0000158644 gene, an homolog of AT3G14840.2 annotated as Leucine-rich repeat trans-membrane protein kinase. The two others have a translational inhibition effect on MDP0000300617 and MDP0000735861, homologues of AT4G18760.1 a receptor like protein 51 (RLP51) involved in signal transduction in plasma membrane and AT5G36930.2, a disease resistance protein of TIR-NBS-LRR class family involved in signal transduction, defense response and apoptosis, expressed in different growth stages, respectively.

The third QTL region investigated was located at the top of LG9. This region spanned 4.8Mb and contained more than 1.100 predicted genes and 21 TF. Among these we identified a TF homologous to SEPALLATA 1 (SEP1) and three copies of SEPALLATA2 (SEP2), among the four found in apple reference genome, and also known as AGAMOUS-LIKE 4 and 2, respectively (AGL4 and AGL2). Another member of the large AGL family, AGAMOUS-LIKE 8 (AGL8), also known as FRUITFULL, was identified in this genomic region. AGL8 is involved in a complex regulatory network in which ASSYMETRIC LEAVES (AS1), another TF involved in leave and gynoecium patterning plays a major role. One TF homologous to AS1 was also present in this genomic region.

Within the confidence interval of the LG9 QTL we identified seven copies of the Senescence-Associated-Genes SAG101, among the 18 copies found in the reference genome, and 3 genes homologous to GLIP7 which function is similar to SAG genes. In addition, several genes potentially involved in the glucose/cellulose biosynthesis pathways were identified: these include 17 glycosyl-transferases and two galacturonases genes. Several auxin transporters of ABCB family (ABCB19 and PGP17) and expansins were also identified.

## Discussion

The detailed phenotyping performed in the present study provides a global view of the relationships between the bearing status of the trees, in relation with alternative bearing behaviour, fruit set, and the maintenance *versus* abscission of young fruits within inflorescences in a range of genotypes. Our results confirm the interdependence among processes occurring at local (inflorescences) and global (whole tree) scales, which are likely to rely on the nutritional status of the young developing fruits. In particular, we found that the mean number of fruits per inflorescence was higher in the ‘on’ year, when the trees were in a high crop load status (Fig. 1, Table 4). This result may appear counterintuitive with respect to the usual acceptance that fruit bud density is negatively correlated to fruit set and to the number of fruits per spur [40] [41]. However, these statements rely on results obtained after artificial manipulation of fruit buds or flowers, followed by a readjustment of fruit set within the trees. Rather, in the present study, the bearing status of the tree was represented by the total number of inflorescences per branch, positively correlated to the mean number of inflorescences with one fruit, both these variables contributing to the ‘on’ status of the trees. By contrast, the total number of inflorescences per branch was not correlated to the mean number of fruits per inflorescence and to the percentage of inflorescences with one fruit, these two variables being influenced by the genotype (Table 2 and 3). Finally, variables defined as percentage, such as percentage of inflorescences with one fruit and percentage of fruit set were more appropriate for analysing the genetic control of tree fruiting behaviour than variables dependent on tree sampling.

Fruit set and the number of fruits per inflorescence were observed on different shoot types. The three categories correspond to different number of leaves and leaf areas, and consequently to different ratio between number of fruits and number of leaves, which has been considered as a key factor for fruit set, development and final quality [42] [43]. As found for year factor, shoot type had a strong effect on the studied variables. Among the shoot types, the lowest year effect was observed on short shoots. This shows that this shoot type is the most suitable for highlighting genotypic differences. It demonstrates that short shoots are less sensitive to environmental effects, and thus may be the most suited for further study of this character on other progenies or elite hybrids under selection.

From a genetic point of view, the total number of inflorescences per branch appeared not affected by the genotype. This probably results from the fact that this number depends on the size of the sampled branches rather than on the genotype. As a consequence, its interest relies on its capability to represent the bearing status of the tree which necessitates counting variables [44] [45]. Other variables such as fruit set and percentage of inflorescences with one fruit were more impacted by the genotype. Because all these variables were not normally distributed, it would have been justified to use generalized mixed model [46]. However, such models are particularly difficult to parametrize, estimate and interpret and we finally decided to estimate the genetic effect with a standard linear mixed model. As a consequence, we also preferred using the mean genotypic values for QTL detection rather than the BLUP, even though QTL detection was performed with both types of phenotypic variables. The heritability values estimated from these models were relatively moderate when compared to values previously obtained for resistance [47], fruit quality [48] [49] or architectural variables [50] [51] [52] [53]. However, the heritability values estimated here are specific to this segregating population, and may not reflect the heritability of this trait overall.

Several QTL were detected along the apple chromosomes. Consistently with heritability values more QTL were detected for fruit set and percentage of inflorescences with one fruit than for counting variables such as the number of inflorescences or the number of fruits per inflorescence. However, the percentage of variation explained by these QTL remained low when compared to heritability values. This suggests that the existence of “missing heritability” probably resulting from the complex nature of the self-thinning traits. Often identified in genome wide association (GWA) and genomic selection studies [54] [55], this missing heritability highlights the limits of marker assisted selection and the difficulties to correctly predict the phenotypes in genomic selection.

Moreover, more QTL were identified for the ‘on’ year (2009) than for the two ‘off’ years (2008 and 2010), and for variables measured on short shoots than on medium or long shoots. Surprisingly, the allelic effects were more often due to male (‘Belrène’) alleles rather than to female (X3263) alleles. Since the X3263 parent had less fruits per inflorescence than ‘Belrène’, the QTL can be interpret as resulting from a “negative” impact of the presence of one out of the two ‘Belrène’ alleles, which promoted more fruit set and less inflorescences with one fruit in contrast with X3263 alleles. QTLs with female (X3263) additive effect were located on LG1 and LG9, whereas the QTLs with male additive effect were on LG10 (top and bottom of LG), LG12 and LG16. Several QTLs involved a parental and a dominance effect, or only a dominance allelic effect.

The QTL located at the bottom of LG1 and controlling the number of inflorescences born on medium shoots overlaps with a QTL controlling the vegetative budbreak dates in the same progeny [33], and co-locates with QTLs identified in another progeny, ‘Starkrimson’×‘Granny Smith’ for annual yields or cumulated yields [44] and for the percentage of branching [56]. This zone is also very well-known for resistance traits since it contains the *Rvi6* (*Vf*) major scab resistance gene and a major scab resistance QTL [57] [58]. However, this zone was not further investigated for candidate genes because of its large confidence interval.

The QTL identified on the top of LG9 and controlling the percentage of inflorescences with one fruits in the ‘ON’ year co-localized with a major QTL controlling vegetative and floral budbreak in the same progeny, as well as the green point’ variable in both ‘Starkrimson’×‘Granny Smith’ and ‘X3263’×‘Belrène’ progenies [33]. This zone was also identified as controlling vegetative budbreak in other progenies [59]. Among few QTL studies that have been performed on fruit abscission on other crops, most of them dealt with abscission close to harvest, e.g. [60] on rice or [61] on melon. [60] noticed that the QTL zone involved in rice grain abscission coincides with a QTL previously detected for seed dormancy and that could belong to a domestication-related block of genes.

### Candidate Gene Identification

Two major approaches are commonly used to dissect complex and quantitative traits, i.e. genome-wide scanning and candidate gene approach. In this study, we identified chromosomal regions controlling the quantitative traits and performed a candidate gene approach using only those portions of the genome located within the confidence interval of three major QTLs. However, despite the use of both approaches, the practicability of our candidate gene approach is limited by its reliance on the first version of the apple genome. Recent findings indicate that up to 15% of contigs, and therefore of predicted genes, might be misplaced on the ‘Golden Delicious’ genome. Therefore, our identification of putative candidate genes is subject to caution, and might not be exhaustive. Furthermore, the candidate gene approach is also limited by its reliance on the known or presumed biology of the phenotype under investigation. In this study, we focussed our attention on genes and TF and miRNA which have been shown to be potentially involved in sugar synthesis and transport, auxin and GA regulation and transport, as well as fruitlet abscission zone activation and development.

The genomic region identified on LG9 contained several TF involved in flower and fruit development, and was particularly rich in AGAMOUS-like family (AGL4, AGL2, and AGL8) genes. Both AGL2 and AGL4 TF have been recently described for their main role in apple fruit development and ripening, especially in hypanthium tissues [62]. AGL8 has been shown to be involved in the coordinated growth of cell tissues and, interestingly, its absence was shown to block silique elongation after fertilization in *Arabidopsis thaliana* [63]. This region also contains putative candidate genes potentially involved in abscission zone development such as glycosyl-transferases, galacturonases, expansines and auxin transporters of the ABCB family [64].

As most members of Senescence-Associated-Gene (SAG) family, SAG101 is involved in plant defenses and responses to stress, and more specifically in responses to wounds and pathogen attacks [65]. Induced by sugars, it is mainly expressed in leaves but also in flowers in *Arabisopsis thaliana* (tair). We could hypothesize that in fruitlets with poor nutrient and sugar supply, expression of this gene might be activated during the abscission process, making this gene a potential marker of fruitlet abscission.

The two genomic region located on LG10 appeared particularly important for fruit set, and for the percentage of inflorescences with fruit. The genomic region located at the top of LG10, near CH04c06 and CH02b07, was the most important for fruit set variables, which all co-localised in this region. This region also co-localized with a QTL previously detected for the ‘green point’ variable in a ‘Starkrimson’×‘Granny Smith’ population [33]. Within this region we identified a putative candidate gene homologous to the TF VND7. In poplar, this TF was shown to be implicated in secondary cell wall biosynthesis and to be a master regulator of xylem vessel element differentiation in poplar [66] [67]. We could hypothesize that, in apple, the expression of this TF might vary among fruitlets of the same inflorescence, and might lead to differences in water supply among those fruitlets. We also identified genes homologous to SUC1 and SUC2, both involved in sugar loading and retrieval in and from the phloem [68]. Variation in the expression of these genes among fruitlets of the same inflorescence could affect sugar supply and this, in turn, possibly leading to differences in fruitlet growth kinetics and to the development of dominant fruits.

Two genes homologous to SCL21 were also identified. These genes belong to the large GRAS family and are known to be involved in a number of developmental processes such as GA responses controlling flowering, shoot and root apex development or xylem patterning [69]. As for VND7, up-regulation of this gene could increase the number of conducting vessels, and lead to varying levels of water supply. SCL21 has been recently described in barley as regulated by miRNA171 and interacting with Phytochrome PAT1 [70].

The second QTL zone located on LG10 in the region of the COL marker is an important genomic region which has previously been detected in many studies involving the columnar trait [51] [71] [72] [73]. Other studies, which did not involve the columnar mutation, also highlighted this zone for other traits such as maximum internode length in specific years [54] and time of vegetative budbreak [33]. Interestingly, this zone also corresponds to a number of QTLs controlling fruiting behaviour and regularity in a ‘Starkrimson’×‘Granny Smith’ progeny, including precocity, number of seeds per fruit, and Biennal Bearing Index (BBI) [44]. Several TF were identified within this QTL region, such as AGL12. In Arabidopsis thaliana, AGL12 was found to contribute to tissues and organ differentiation, especially floral organs [74], and more specifically in late flowering [75]. Recently, AGL12 was found to be specifically expressed in the abscission zone (AZ) in conjunction with SHOOT MERISTEMLESS (STM), also present in the zone, which was down-regulated during abscission of tomato fruit [76]. Both genes might have a strong effect on apple fruitlet self-thinning. Among the WRKY family [77], WRKY65 has been shown to be involved in senescence triggering after sugar starvation [78]. We could hypothesize that this gene might be expressed in some fruitlet pedicels following a decreased sugar supply, thus triggering senescence of tissues and leading to fruitlet drop. Other genes were also identified in this QTL region, including two putative trehalose synthetases. Trehalose is a disaccharide formed by a 1,1-glucoside bond between two α-glucose units, and is an effective signal molecule that may have an essential role in developmental processes [79]. Variation in trehalose synthase expression in fruitlets of a same inflorescence might thus be important for fruit development. Finally, a gene with sequence similarity to IRX1 was identified within the confidence interval of this QTL. IRX1 was shown to be involved in secondary cell wall biosynthesis. In *Arabidopsis thaliana*, the loss of IRX1 function provokes mutants with abnormal xylem formation, reduced cellulose content, and enhanced drought and osmotic stress tolerance [80].

### Conclusion

In this study, we performed a QTL analysis to study the genetic determinism of complex traits involved in the self-thinning character of young apple fruits. This genetic dissection allowed us to identify moderate to strong QTLs using a F1 segregating population, and to screen for putative candidate genes located within the confidence interval of three major QTLs. This investigation allowed us to identify several genes potentially involved in the formation of conducting vessels, the synthesis and transport of sugars and hormones, and finally the activation and development of the abscission zone. Despite its major importance, the self-thinning character of fruitlets is not a common trait in apple, and very few elite cultivars have the ability to induce fruitlet abscission. Thus, the validation of QTLs in additional cycles of alternate bearing and in different genetic background, as well as the identification of new QTLs controlling self-thinning will be of major importance in future studies. Finally, we believe that the identification of QTLs and genes responsible for variations of this character will have a strong impact in future apple breeding programme, and will ultimately contribute to a major reduction in the use of thinning reagents by growers.

## Supporting Information

Table S1 **Length of SSR alleles (in bp) at loci associated with putative QTLs and corresponding code for map construction.**
(DOC)

Table S2 **List of genes in the three QTL regions selected for putative candidate genes screening.**
(XLS)

Table S3 **MIR accessions corresponding to the three QTL regions selected for putative candidate genes screening.**
(XLS)

Table S4 **Predicted miRNA/Target Pairs corresponding to the three QTL regions selected for putative candidate genes screening.**
(XLS)
