# Supplementary material for: Fruit Self-Thinning: A Trait to Consider for Genetic Improvement of Apple Tree
Source: PLoS One. 2014 Mar 13;9(3):e91016. doi: 10.1371/journal.pone.0091016 (PMC3953208; doi:10.1371/journal.pone.0091016)
Supplement: Table S1 — Length of SSR alleles (in bp) at loci associated with putative QTLs and corresponding code for map construction. (DOC) [file pone.0091016.s001.doc]

Supplementary Table 1. Length of SSR alleles (in bp) at loci associated with putative QTLs and corresponding code for map construction

| Cofactor | LG | X3263 | Belrène | Code |
| --- | --- | --- | --- | --- |
| Hi07d08 | 1 | 206 - 248 | 206 - 238 | <efxeg> |
| GD142 | 9 | 154 - 160 | 162 - 166 | <abxcd> |
| COL_XB | 10 | 240 - 248 | 250 - 252 | <abxcd> |
| GD_SNP01867 | 10 | - | CC | <lm x ll> |
| ch02b07_XB | 10 | 105 - 126 | 103 - 126 | <efxeg> |
| ch04a12_XB | 11 | 177 - 179 | 194 - 196 | <abxcd> |
| GD_SNP01769 | 12 | CT | CC | <lmxll> |
| ch02c09 | 15 | 249 - 261 | 271 - 273 | <abxcd> |
| Hi08f12 | 16 | 111 - 156 | 127 - 213 | <abxcd> |
